# Clinical implication in the use of the AAA algorithm versus the AXB in nasopharyngeal carcinomas by comparison of TCP and NTCP values

**DOI:** 10.1186/s13014-020-01591-7

**Published:** 2020-06-12

**Authors:** Antonella Bufacchi, Orietta Caspiani, Giulia Rambaldi, Luca Marmiroli, Giuseppe Giovinazzo, Mattia Polsoni

**Affiliations:** 1 Medical Physics Department, S. Giovanni Calibita Fatebenefratelli Hospital – Amethyst Radioterapia Italia and PIOXI Clinic, Rome, Italy; 2 U.O.C. Radiotherapy, S. Giovanni Calibita Fatebenefratelli Hospital – Amethyst Radioterapia Italia, Rome, Italy; 3 Medical Physics Department, S. Giovanni Calibita Fatebenefratelli Hospital – Amethyst Radioterapia Italia, Rome, Italy; 4 Amethyst Radioterapia Italia, Rome, Italy

**Keywords:** Dose volume histogram, Tumor control probability, Normal tissue complication probability, Anisotropic analytical algorithm, Acuros XB algorithm

## Abstract

**Purpose:**

Retrospective analysis of volumetric modulated arc therapy treatment plans to investigate qualitative, possible, clinical consequences of the use of AAA versus AXB in nasopharyngeal cancer (NPC) cases.

**Methods:**

The dose distribution of 26 treatment plans, produced using RapidArc technique and AAA algorithm, were recalculated using AXB and the same number of monitor units provided by AAA and clinically delivered to each patient. The potential clinical effect of dosimetric differences in the planning target volume (PTV) and in organs at risk (OAR) were evaluated by comparing TCP and NTCP values. The Wilcoxon Signed Rank test was used for statistical comparison of all results obtained from the use of the two algorithms.

**Results:**

The poorer coverage of the PTV, with higher prescribed dose, was reflected in the TCP, which was significantly lower when AXB was used, the median value was 81.55% (range: 74.90, 88.60%) and 84.10% (range: 77.70, 89.90%) for AAA (*p* < 0.001). OAR mean dose was lower in the AXB recalculated plan than the AAA plan and the difference was statistically significant for all the structures. The NTCP for developing mandible necrosis showed the largest median percentage difference between AAA and AXB (56.6%), the NTCP of risk for larynx edema of Grade ≥ 2 followed with 12.2%.

**Conclusions:**

Differences in dose distribution of NPC treatment plans recalculated with AXB are of clinical significance in those situations where the PTV and OAR involve air or bone, media in which AXB has been shown to more accurately represent the true dose distribution. The availability of AXB algorithm could improve patient dose estimation, increasing the data consistency of clinical trials.

## Background

Treatment of head and neck cancers using Intensity Modulated Radiation Therapy (IMRT) or Volumetric Modulated Arc Therapy (VMAT) is a promising technique due to its ability to conform high dose to irregularly shaped volumes and to steer doses away from multiple critical normal organs. However more demanding modern treatment techniques require better modeling of treatment beams and more sophisticated modeling in the presence of inhomogeneities in order to guarantee accuracy in the calculation of dose distribution.

Advanced (‘type b’) dose calculation algorithms (such as AAA – Anisotropic Analytical Algorithm) now routinely available in commercial treatment planning systems show improved accuracy compared to the previous pencil beam (‘Type a’) algorithms, accounting for lateral electron transport, but some errors still persist. The convolution-superposition algorithm, the AAA and the collapsed cone convolution algorithm (type-b algorithms) were proved to significantly overestimate the doses near air/tissue interfaces [1–4].

The nasopharyngeal carcinomas region is surrounded by a considerable amount of bony structures and air cavities, the limitations of the algorithms mentioned above may affect the reliability of the calculated dose distribution.

The Acuros XB (AXB) algorithm, recently introduced in the Eclipse treatment planning system (Varian Medical Systems, Palo Alto, USA) [5] accounts for the effects of heterogeneities in patient dose calculation by explicitly solving the linear Boltzman transport equation (LBTE) that describes the macroscopic behavior of the radiation particles as they travel through and interact with matter. Some recent investigations have shown that AXB is able to achieve comparable accuracy to the golden standard of Monte Carlo calculations in heterogeneous media [6–8].

Previous studies quantified the difference between the use of AXB vs AAA for calculating dose for breast, lung and nasopharyngeal cancer treatments.

For breast cancer treatments, Fogliata et al. [9] show how the analysis of the two breast structures presenting densities comparable with muscle and with adipose tissue showed an average difference in dose between AXB and AAA of 1.6%, with AAA predicting higher dose than AXB, for muscle tissue (the lobular breast), while the difference for adipose tissue was negligible.

For Non-Small-cell lung cancer treatments, again Fogliata et al. [10] investigated the clinical impact of the AXB. The planning target dose difference was stratified between the target in soft tissue, where the mean dose was found to be lower for AXB with a range of 0.4 to 1.7%, and the target in lung tissue, where the mean dose was higher from 0.2 to 1.2% for 6MV and lower for 15 MV up to 2.0%.

Studies Kan et al. [11] carried out for nasopharyngeal carcinomas treatments show how when using AXB instead of AAA, the averaged mean dose to PTV was found to be up to 1.2% lower and the averaged minimum dose to PTV in bone was 4% lower, whereas it was 1.5% lower for PTV in tissue.

Interesting is the investigation of the radiobiological impact of AXB compared to AAA in treatment planning. For lung cancer treatments the impact of the dose distribution differences on the NTCP of the lungs and the heart was reported [12, 13]. For whole breast cancer treatments, Petillion et al. [14] show how the more advanced algorithms predicted a significantly lower TCP and NTCP for moderate breast fibrosis; the differences varied between 1 and 2.1% for TCP and between 2.9 and 5.5% for NTCP. In the study of Padmanaban et al. [15] compared to the AAA algorithm, the AXB was found to significantly alter the tumor control probability (TCP) for treatment of oesophageal cancer.

Studies on the radiobiological impact for nasopharyngeal cancer (NPC) treatments due to the recalculation of dose distribution using AXB instead of AAA are lacking; bringing up this subject is interesting because the target volumes include a considerable amount of air cavities and bony structures. We, therefore, investigated the radiobiological impact (both on the TCP and on the NTCP) in NPC patients treated with VMAT.

## Methods

### Patient data, treatment planning and delivery technique

Twenty-six clinical treatment plans of NPC patients with stages I trough IV were reviewed for this study.

The target volume of each patient was defined by oncologist in charge using 1.25 mm thick axial CT images. The gross tumor volumes (GTV) included all known gross disease as determined by imaging and clinical findings. The margins were adjusted to 1.0 cm beyond the GTV to obtain the CTV; the CTV was expanded symmetrically by 0.3 cm in all directions to account for patient setup and motion within the thermoplastic mask.

The prescribed doses were 69.96 Gy to high-risk PTV (PTV_1_), 59.40 Gy to intermediate-risk PTV (PTV_2_) and 54.45 Gy to low-risk PTV (PTV_3_) with simultaneous integrated boost in 33 fractions. The patients were irradiated with RapidArc (RA) treatments, VMAT with two complete arcs with collimator 10° and 350°, respectively, plus one complete arc with collimator 0°. All plans were generated using a 6 MV beam and modulated with a 120 multileaf collimator from a linear accelerator (Truebeam – Varian Medical Systems, Palo Alto, USA).

The treatment plans were developed using Eclipse 15.5 TPS (Treatment Planning System); the dose distributions of the clinical treatment plans initially performed using the AAA algorithm were recalculated with AXB using the same number of monitor units provided by AAA. Dose to medium calculation was selected for Acuros XB, accounting for the element composition of specific anatomical regions as derived by the CT dataset. Tissue segmentation was automatically performed based on density ranges derived from the HU values read in the CT dataset of the patients. For each tissue, the specific chemical composition was based on the ICRP Report 23 [16].

By the visual inspection of the isodose distribution and DVHs, a treatment plan was deemed satisfactory if certain normal tissue dose criteria were met and the isodose lines indicated a “good” tumor coverage. Usually one tried to ensure that the degree of heterogeneity was kept within + 7% and − 5% of the prescribed dose in accordance with the ICRU Report 62 [17].

Data were tested for normality with the Shapiro-Wilk test and different datasets were compared with the Wilcoxon Signed Rank test. A *p* value < 0.05 was considered the threshold for statistical significance.

For the validation of both the algorithms implemented in the TPS, the tests, the analysis, and the acceptability criteria were in large part based on the report of the AAPM Report 55 [18], other documents such as the technical report by IAEA [19] were consulted. For AAA and AXB, the outcomes of some test were comparable to those provided by Van Esch et al. [20] and Fogliata et al. [21], respectively.

### NTCP and TCP analysis

The NTCP was evaluated by applying different radiobiological models according the analyzed endpoints. To take dose fractionation into account, dose-volume histograms (DVHs) were corrected to 2 Gy/fraction equivalent (LQED2) [22], assuming a α/β value of 3 Gy.

For quantifying the risk of xerostemia from irradiation of the parotid glands, of developing grade ≥ 2 laryngeal edema, of mandible necrosis and myelophathy, the NTCP was calculated using Lyman Kutcher-Burman (LKB) model [23–25] (details on the model are given in Appendix). The applied parameters are listed in Table 1.

**Table 1:** Summary of NTCP modeling studies (SWALM6: physician-rated swallowing dysfunction 6 months after (CH) RT)

LKB model parameters					
OAR	Reference	LKB parameters			Endpoint
Parotid glands		n	D_50_ (Gy)	m	
	Eisbruch et al. [26]	1.00	28.40	0.18	25% xerostomia at 1 year
	Roesink et al. [27]	1.00	39.00	0.45	25% xerostomia at 1 year
Mandible	Burman et al. [25]	0.07	72.00	0.10	necrosis
Larynx	Rancati et al. [28]	1.17	47.30	0.23	grade ≥ 2 edema
Spinal cord	Kirpatrick et al. [29], Emami et al. [30]	0.07	72.00	0.10	myelophathy
NTCP = (1 + e^-S^)^− 1^					
**OAR**	**Reference**	**S**			**Endpoint**
Thyroid gland	Boomsma et al. [31]	0.011 + (0.062*D_mean_) + (− 0.19*V)			hypothyroidism
PCM and supraglottic larynx (SL)					
	Christianen et al. [32]	−6.09 + (D_mean(PCM)_ *0.057) + (D_mean(SL)_ *0.057)			SWALM6
	Christianen et al. [32]	−6.89 + (D_mean(PCM)_ *0.049) + (D_mean(SL)_ *0.048) + (age*0.795)			problems with swallowing solid food

To calculate the NTCP and the TCP, the DVHs were imported to Biosuite (Clatterbridge Cancer Center, Bebington, Wirral, UK) [33].

The following equation [34]:

1 $$ NTCP={\left(1+{e}^{-S}\right)}^{-1} $$

was used to calculate the risk of radiation-induced hypothyroidism.

Soproglottic larynx and superior pharyngeal constrictor muscle (PCM) were also contoured except for three patients where the surgical intervention was so invasive to make impossible to delineate these contours. NTCP for physician-rated swallowing dysfunction 6 months after (CH) RT (SWALM6) (primary endpoint) and for the secondary endpoint concerning the swallowing solid food dysfunction was performed by Eq. (1).

The values of S parameter are reported in Table 1.

Using the LQ model, the TCP was calculated from DVHs of the PTV_1_. The radiobiological parameters used in the model were derived from the study by Lee et al. [35]: the values of α and α/β were taken as 0.33 Gy^− 1^ and 10 Gy, respectively; a clonogenic cell density of 10^7^ cells/cm^3^ was assumed [36].

### Dose analysis

For the PTV_1,2,3_, we evaluated D_95%_, D_2%_ dose levels on the DVH above which lay 95 and 2% of the volume of the PTV_1_; they were used as a surrogate for dose minimum and dose maximum, respectively. The mean dose (physical dose) to the PTV_1,2,3_ was also recorded.

The mean dose was assessed for all OARs; for spinal cord and mandible, because their structure predominately serial, D_2%_, was also considered.

## Results

**Fig. 1:**
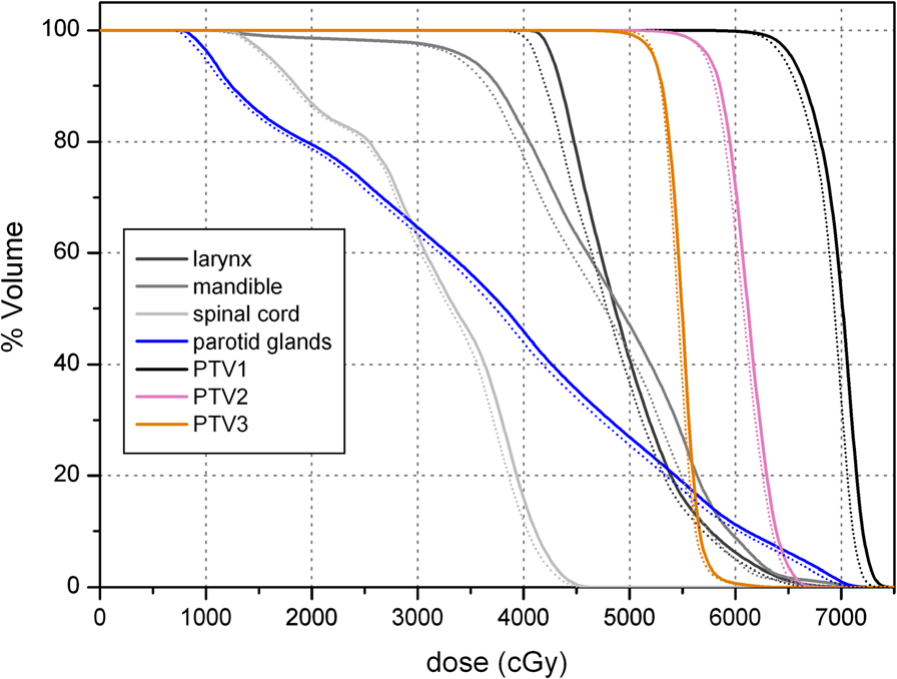
Example of a comparative DVH for a NPC plan. The curves calculated by the AAA algorithm are depicted by solid lines and those calculated by AXB by dotted lines

The results of the comparison of the treatments plans as calculated by two algorithms, AAA and AXB, are summarized in Tables 2, 3. A comparison of the total physical dose DVHs of the PTV_1,2,3_ and OARs for a typical patient plan calculated using the two dose algorithms is shown in Fig. 1.

**Table 2:** Comparison of dose to PTV_s_ calculated using AAA and AXB for all patients

Target (dose metric)	Median dose [min,max] in Gy		*p*
	AAA	AXB	
PTV_1_(D_95%_)	66.8 [64.1,69.1]	65.8 [62.9,68.3]	< 0.001
PTV_1_(D_2%_)	72.7 [70.6,73.6]	72.0 [70.0,73.2]	< 0.001
PTV_1_(D_mean_)	70.2 [68.1,71.1]	69.5 [67.3,70.9]	< 0.001
PTV_2_(D_95%_)	58.1 [53.0,64.5]	57.9 [54.8,65.6]	**0.310**
PTV_2_(D_2%_)	66.0 [62.1,70.7]	65.6 [61.5,70.2]	0.008
PTV_2_(D_mean_)	61.3 [57.9,68.0]	60.8 [57.6,67.3]	< 0.001
PTV_3_(D_95%_)	52.4 [50.4,56.6]	52.1 [50.1,56.4]	**0.266**
PTV_3_(D_2%_)	58.9 [55.5,71.3]	58.8 [55.7,69.8]	0.03
PTV_3_(D_mean_)	55.1 [52.8,61.0]	54.66 [52.7,60.4]	< 0.001

**Table 3:** Median and range of D_mean_ and D_2%_ to OAR estimated by AAA and AXB over all patients

OAR (dose metric)	Median dose [min,max] in Gy		*p*
	AAA	AXB	
larynx (D_mean_)	43.5 [33.0,63.2]	42.7 [32.1,62.2]	< 0.001
mandible(D_2%_)	70.1 [46.0,73.1]	67.8 [44.7,71.2]	< 0.001
mandible (D_mean_)	47..3 [25.1,58.6]	45.7 [24.4,56.6]	< 0.001
parotid glands (D_mean_)	29.2 [19.8,48.5]	28.3 [19.1,47.6]	< 0.001
superior PCM (D_mean_)	63.4 [49.0,68.1]	62.8 [48.4,68.3]	< 0.001
spinal cord(D_2%_)	37.7 [23.1,43.8]	37.0 [22.3,43.1]	< 0.001
spinal cord (D_mean_)	27.2 [18.4,34.1]	26.6 [17.8–33.1]	< 0.001
supraglottic larynx (D_mean_)	45.8 [35.3,69.2]	44.9 [34.3,68.1]	< 0.001
thyroid (D_mean_)	54.1 [38.7,64.3]	53.0 [37.9,63.4]	< 0.001

Subsequently, NTCP calculated with AAA and AXB algorithm are referred to as NTCP_AAA_ and NTCP_AXB_ respectively; the NTCP values less than 0.1% are assumed to be zero.

### Dose to PTV and TCP

It appears that lower doses for D_95%_, D_2%_ and D_mean_ in the re-calculated AXB plans, as compared to AAA plans (Table 2).

When AXB was used, the median percentage difference for D_95%_, D_2%_ and D_mean_ of PTV_1_ were reduced by 1.5% (range: 0.1, 4.0%; *p* < 0.001), 0.8% (range: 0.3, 1.8%; *p* < 0.001) and 1.1% (range: 0.1, 1.4%; *p* < 0.001). For PTV_2_ and PTV_3_ the results, regarding D_2%_ and D_mean_, were similar to PTV_1_, while for D_95%_ the difference was not statistically significant. The more reduction in D_95%_ was observed in PTV_1_ that generally encompassed a more high portion of bony structures, such as mandible, cervical vertebrae and skull base.

**Fig. 2:**
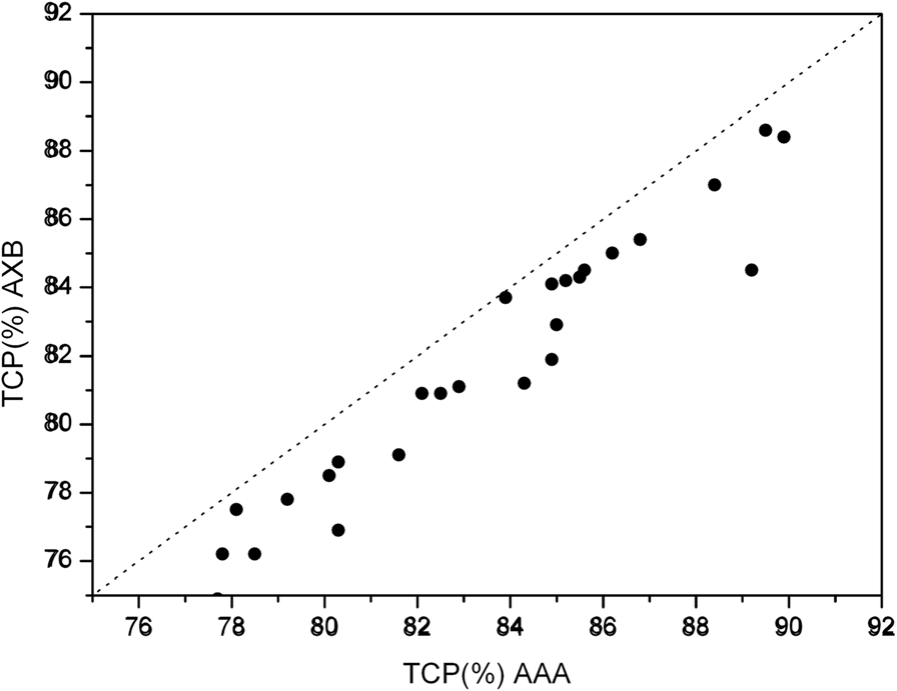
Comparison of TCP for PTV_1_ computed with the AAA (abscissa) and the AXB (ordinate) algorithm. Each symbol represents data of an individual patient. The dotted line indicates the line of *identity*

**Fig. 3:**
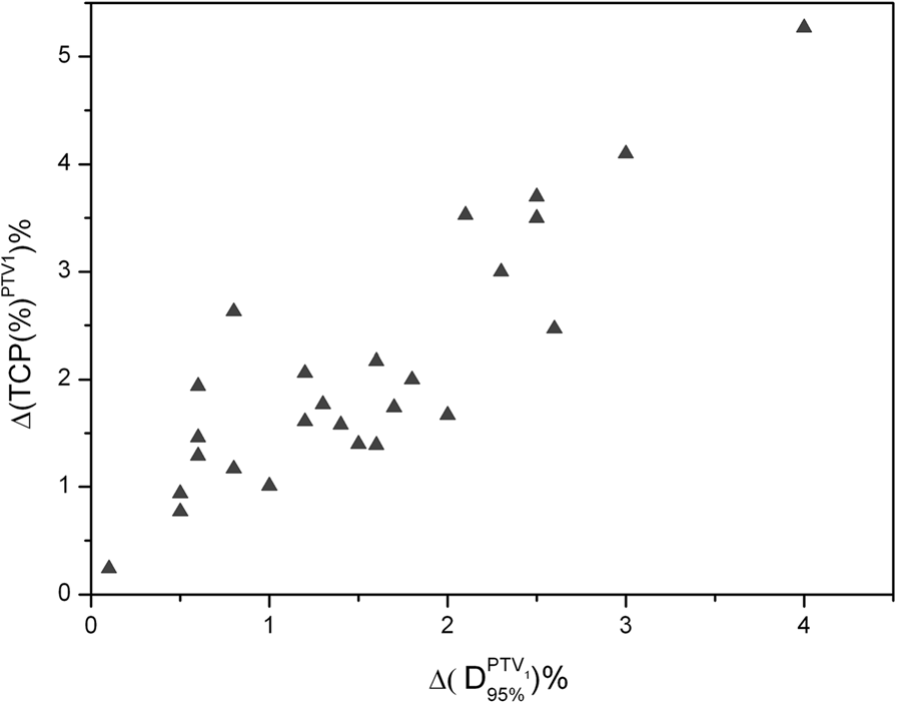
Δ(TCP)% (AAA vs AXB) versus Δ(D_95_)% (AAA vs AXB) regarding PTV_1_

The poorer coverage of the PTV_1_ was reflected in the TCP, which was significantly lower when the AXB was used, the median value was 81.55% (range: 74.90, 88.60%) and 84.10% (range: 77.70, 89.90%) for AAA (p < 0.001) (Fig. 2). Figure 3 shows the percentage TCP difference between AAA and AXB plans (ΔTCP%) versus the percentage D_95%_ differences in the AAA and AXB plans (ΔD_95%_%). It clearly shows that ΔTCP% increases as ΔD_95%_%. The percentage TCP difference can be as large as 5.3% on the case with a 4.0% percentage difference in D_95_.

### Dose to OARs and NTCP

The maximum percentage difference for D_mean_ of OARs, averaged over the 26 patients, was 3.4% for the mandible; the minimum percentage difference was 0.9% for PCM. The difference between the two algorithms in terms of D_mean_ to OARS was statistically significant for all the structures.

The percentage difference for D_2%_ of mandible and spinal cord were 3.1 and 1.9% respectively.

Interestingly, the Eisbruck et al. [26] parameters predicted much higher NTCP value for the risk of a decrease in the salivary flow to 25% of the pre-treatment flow at 1 year post treatment than the risk calculated by Roesink et al. [27] parameters which considered the same endpoint (see Table 1). This is because the Eisbruck et al. parameters used a much shaper slope of the response curve compared with the other parameter set, which results in a more dose-sensitive NTCP prediction. -9.3% and − 5.1 was the percentage difference between the median NTCP_AXB_ and NTCP_AAA_ value when Eisbruck et al. and Roesink et al. parameters were applied respectively.

The risk for developing mandible necrosis was found to be much higher when the AAA was used, an increase of 56.6% was observed: median NTCP 6.5% (range: 1.8, 31.8%) vs 2.8% (range: 0.5%, 17.7) when AXB was used.

Regarding the larynx, the use of AAA resulted in a median D_mean_ equal to 43.5 Gy (range: 33.0, 63.2 Gy) vs 42.7 Gy (range: 32.1 Gy, 62.2 Gy) for AXB. The median NTCP_AXB_ of risk for larynx edema of Grade ≥ 2 was significantly lower than NTCP_AAA_: 19.2% (range: 2.4–72.6%) vs 21.8% (range: 3–75.2%); the percentage difference was 12.2%.

− 1.9, − 1.7 were the percentage difference between AXB and AAA for the median of thyroid gland D_mean_ and NTCP for developing hypothyroidism respectively; the difference were statistically significant.

D_mean_ to superior pharyngeal constrictor muscle (PCM) and supraglottic larynx were recorded for both plans developed with AXB and AAA. Moderate percentage difference (though statistically significant) between AXB and AAA were seen for the median value: − 0.94% and − 1.9% for PCM and supraglottic larynx respectively. For SWALM6 the median NTCP_AXB_ value was 31.7% (range: 20.4, 54.2%) vs 33.1% (range: 21.5, 55.7%) for NTCP_AAA_; it resulted in a percentage difference of − 4.2% and the median of the percentage differences between NTCP values, Δ(NTCP)%, across the whole patient population was 4.1% (range: 2.9, 7.4%).

For the secondary endpoint, the median NTCP_AXB_ was 28.1% (range: 11.6, 58.4%) vs 29.2% (range: 12.4, 59.9%) for NTCP_AAA_ and the median of the percentage differences between NTCP values, Δ(NTCP)%, across the whole patient population was 4.5% (range: 2.5, 6.9%).

The incidence of myelophathy predicted by available parameters set was zero, but on the other hand all the plans respected the maximum dose to spinal cord which was inferior to 46 Gy.

## Discussion

Previous studies investigating the use of AXB in heterogeneous media suggest that this algorithm is more accurate than the widely-used AAA. Consequently the comparison between AXB and AAA dose distribution by analysed dose indices, provides an indication of the difference between the dose predicted by the AAA and that considered as a better approximation of true delivered dose. In our study, we showed that the photon dose calculation algorithm used in NPC treatments has radiobiological and, therefore, clinical impact. This study quantifies the radiobiological impact of the differences between the physical dose distributions in NPC by NTCP and TCP.

The differences in dose to target predicted by two algorithms are of a magnitude such that the choice of algorithm has clinical impact: the TCP percentage difference can be up to 6.8%. Normalization of treatment plans using AXB to meet the protocol dose prescription of 69.96 Gy would result in an increase in MU of around 1.7% (range 1.0 to 2.2%) with a corresponding increase in dose delivered to the OARs. More radiation output to produce the same coverage as AAA involves a corresponding increase in dose delivered to the surrounding OAR.

This is in line with results reported in the study by Kan et al., mentioned in background section.

**Fig. 4:**
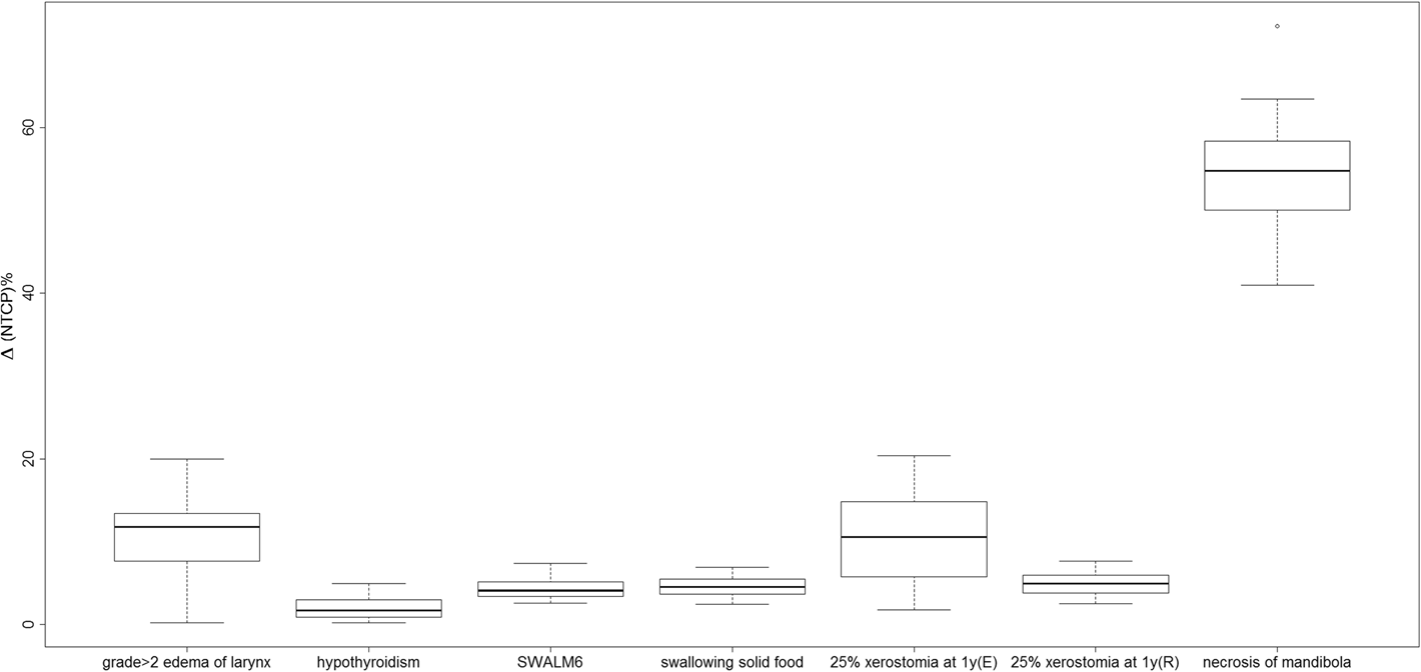
Box plot of Δ(NTCP)% (AAA vs AXB) for the different endpoints. The bold line represents the median of the percentage difference and the black bars represent the range of the data. (*R* and *E* refer to Roesink et al. and Eisbruch et al. parameters, respectively)

Figure 4 shows the box plot of the percentage ΔNTCP (AAA vs AXB) comparison between the different OARs. The NTCP for developing mandible necrosis shows the largest median ΔNTCP (56.6%), the NTCP of risk for larynx edema of Grade ≥ 2 follows with percentage ΔTCP equal to 12.2%. For the other OARs, the percentage ΔΝTCP is lower than 5%, except for Eisbruck et al. parameters that is able to show better discriminate between the dose calculations algorithms.

The AXB calculates dose considering the element composition; unlike most water-like tissue in body, such as muscle and lung, the elemental composition of compact bone (such as mandible) is very different from that of water. Siebers et al. [37] reported that dose calculations neglecting the element composition resulted negligible effect in lighter tissue but not in compact bone. Consequently our results found the largest differences in PTVs and OARs containing bony.

Regarding the larynx, it is a structure surrounding air; AXB shows a better agreement with Monte Carlo calculation [38] in regions of re-buildup in soft tissue after the beam has passed through low density tissue such as air and therefore lower doses beyond the air/tissue interface than AAA along the central axis. This effect of dose reduction in air and near air/tissue interfaces appears responsible for higher ΔNTCP of risk for larynx edema of Grade ≥ 2 compared with the remaining ΔNTCPs.

The comparison of the two algorithms in the present study is in accordance with the literature; in NPC treatments, the differences are of minor clinical significance in some situations such as when the PTVs and OAR don’t involved air or bone. The adoption of the AXB into clinical treatment planning practice requires one to fully understand its effect and its potential consequences so as to re-evaluate an assessment of dose-effect relationships and of parameters used in treatment planning decisions.

Similarly, the introduction of a predictive model into clinical practice has to be prudent as it is necessary to assess if it is based on calculations and treatments similar to those for which the NTCP has to be calculated. There are large uncertainties in the biological models and its associated parameters; the more accurate dose distribution given by AXB would be useful to have a better understanding of the treatment outcomes. As more clinical data are collected, it may help in the formulation of models to predict radiobiological response and result in more accurate prediction of TCP and NTCP.

The published TCP/NTCP model parameters that we used were obtained from studies that used different techniques and dose algorithms from the present study. Whatever the case, the use of these TCP/NTCP model parameters is appropriate because our study performs a relative comparison between two different dose calculation algorithms rather than studying the absolute expected values.

The results found in this study show how for NPC treatments the differences between the dose distributions of the two tested algorithms yield statistically significant differences in the NTCP and TCP values.

## Conclusion

In this study, we have tried to investigate qualitative, possible clinical consequences of the use of AAA versus AXB (keeping the same number of monitor units provided by AAA and clinically delivered to each patient) for NPC treatments by comparing NTCP and TCP values. As a result, the NTCP_AXB_/TCP_AXB_ was lower than the NTCP_AAA_/TCP_AAA_; the difference could be clinically significant. The availability of AXB algorithm could improve patient dose estimation, increasing the data consistency of clinical trials. This could improve radiobiological models and obtain more robust radiobiological parameters.

## Data Availability

Not applicable.

## Appendix

### The LKB model

The NTCP for given volume, V, irradiated by an uniform dose, D, is given by following equation:

$$ NTCP:\frac{1}{\sqrt{2\pi }}\underset{-\infty }{\overset{t}{\int }}\exp \left(\frac{-{x}^2}{2}\right) dx $$

where

$$ t=\frac{D-{D}_{50}\left(\mathrm{v}\right)}{m\ast {D}_{50}\left(\mathrm{v}\right)} $$

$$ {D}_{50}\left(\mathrm{v}\right)={D}_{50}(1){\mathrm{v}}^{-n} $$

$$ v=\frac{v}{v_{ref}} $$

*m* is a dimensionless parameter that represents the steepness of the dose-response curve, D_50_(1) is the dose tolerance of an organ at which there is 50% complication probability, D_50_(ν) is the dose tolerance for a partial volume *ν*. *n is* the parameter that determines volume-dependence of the complication in the organ: *n = 0* indicates that the organ has a serial structure and the maximum dose determines the complication probability whereas *n = 1* indicates a parallel structure in which the mean dose is the predictor of the complication probability.

### Tumor Control Probability (TCP)

Using the LQ model, the TCP calculated for the entire *V* volume irradiated uniformly with dose *D* can be expressed as follows:

$$ TCP\left(\mathrm{V},\mathrm{D}\right)=\exp \left\{-K\exp \left[-\left(\alpha +\beta d\right)\mathrm{D}\right]\right\} $$

Where *K* is the number of clonogenic cells, α and β are tissue specific parameters related to cell radiosensitivity (they are expressed in units Gy^− 1^ and Gy^− 2^, respectively), d is dose per fraction.

When the dose in the V volume is nonuniform, its distribution must be taken into account. A standard way to condense the dose distribution data in the V volume is to use the differential dose-volume histogram (dDVH), where the dose range is divided into M bin values and for each bin value D_j_, the sum volume ν_j_ of all voxels receiving the dose D_j_, is calculated. Indicating the fraction of volume ν_j_/V with ε_j_, the dDVH is expressed by the set of M couples {( ε_j_, D_j_)} with *j* = 1,…,M and TCP can be calculated as [39]:

$$ TCP\left\{\left({\varepsilon}_j,{D}_J\right)\right\}=\left[ TCP\left(V,D\right)\right] $$

The F is expressed as:

$$ F=\sum \limits_{j=1}^M{\varepsilon}_j\exp \left[-\left(\alpha +\beta {d}_j\right){D}_j+\left(\alpha +\beta d\right)D\right] $$

## Abbreviations

AAA: Anisotropic analytical algorithm
AXB: Acuros XB algorithm
DVH: Dose volume histogram
IMRT: Intensity Modulated Radiation Therapy
VMAT: Volumetric Modulated Arc Therapy
TPS: Treatment planning system
OARs: Organs at risk
PTV: Planning treatment volume
NPC: Nasopharyngeal carcinomas
D_x%_: Dose level on the DVH above which lay x% of the observed volume
TCP: Tumor control probability
NTCP: Normal tissue complication probability
